# [Retracted] Silencing of RTKN2 by siRNA suppresses proliferation, and induces G1 arrest and apoptosis in human bladder cancer cells

**DOI:** 10.3892/mmr.2024.13258

**Published:** 2024-05-29

**Authors:** Yi-Xiang Liao, Jin-Min Zeng, Jia-Jie Zhou, Guang-Hua Yang, Kun Ding, Xian-Jue Zhang

Mol Med Rep 13: 4872–4878, 2016; DOI: 10.3892/mmr.2016.5127

Following the publication of this paper, it was drawn to the Editor's attention by a concerned reader that certain of the Transwell migration assay data shown in Fig. 4D on p. 4876 were strikingly similar to data that had already been published in different form in another article written by different authors at a different research institute. In addition, a pair of the data panels in Fig. 4D were overlapping, indicating that data derived from the same original source had been used to represent what were intended to be the results obtained from differently performed experiments.

Owing to the fact that the contentious data in the above article had already been published prior to its submission to *Molecular Medicine Reports*, the Editor has decided that this paper should be retracted from the Journal. The authors were asked for an explanation to account for these concerns, but the Editorial Office did not receive a reply. The Editor apologizes to the readership for any inconvenience caused.

